# On static black holes solutions in Einstein and Einstein–Gauss–Bonnet gravity with topology $\mathbf{S^{n} \times S^{n}}$

**DOI:** 10.1140/epjc/s10052-015-3481-y

**Published:** 2015-06-24

**Authors:** Naresh Dadhich, Josep M. Pons

**Affiliations:** Centre for Theoretical Physics, Jamia Millia Islamia, New Delhi, 110025 India; Inter-University Centre for Astronomy and Astrophysics, Post Bag 4, Pune, 411 007 India; Departament d’Estructura i Constituents de la Matèria and Institut de Ciències del Cosmos (ICCUB), Facultat de Fsica, Universitat de Barcelona, Diagonal 647, 08028 Barcelona, Catalonia Spain

## Abstract

We study static black hole solutions in Einstein and Einstein–Gauss–Bonnet gravity with the topology of the product of two spheres, $\mathbf{S^{n} \times S^{n}}$, in higher dimensions. There is an unusual new feature of the Gauss–Bonnet black hole: the avoidance of a non-central naked singularity prescribes a mass range for the black hole in terms of $\Lambda >0$. For an Einstein–Gauss–Bonnet black hole a limited window of negative values for $\Lambda$ is also permitted. This topology encompasses black strings, branes, and generalized Nariai metrics. We also give new solutions with the product of two spheres of constant curvature.

## Introduction

The study of gravity in higher dimensions was given great impetus by string theory, for which it is a natural framework. One of the most compelling pictures that emerges is that all matter fields are believed to remain confined to the usual 4-dimensional spacetime 3-brane, while gravity can propagate in higher dimensions. At the root of this perception, we believe, is the unique gravitational property of universality – its linkage to all that physically exists. On the other hand it has also been argued by one of us [1–3] purely based on classical considerations, that gravity cannot be entirely confined to a given dimension. High energy effects would require the inclusion of higher orders in the Riemann curvature in the action; then, if the resulting equation is to remain second order, it has to be the Lovelock polynomial which makes non-trivial contributions only in $D>4$. That is, high energy effects could only be realized in higher dimension; see [3, 4] and references therein. It is envisioned as a general guiding principle that anything universal should not be constrained from outside but should rather should be left to itself to determine its own playground. This is precisely what Einstein gravity does. Since it is universal and hence it can only be described by spacetime curvature [1, 2], it is what determines the gravitational law [3]. It is important to note that it is not prescribed from outside like the Newtonian law. Similarly higher dimensions should also be dictated by some property of gravity like the high energy effects. Apart from a strong suggestion, we have not yet been able to identify a gravitational property that asks for higher dimension. In the absence of such a guiding direction, it is a prudent strategy to probe gravitational dynamics in higher dimensions so as to gain deeper insight. We believe this is the main motivation for higher dimensional investigations of gravity.

The first question that arises is: what equation should describe gravitational dynamics in higher dimensions? Should it be the Einstein equation or should it be its natural generalization, the Lovelock equation? The Einstein equation is linear in Riemann, while the Lovelock equation concerns a homogeneous polynomial – yet it has the remarkable property that the resulting equation still remains second order quasilinear. The higher orders in Riemann become relevant only in higher dimensions. If for physical reasons, like high energy effects, higher orders in Riemann are required to be included and the equation should continue to remain second order, the Lovelock equation is uniquely singled out and then requires higher dimensions for the realization of higher order Riemann contributions [1, 2, 4]. The next question is: should the equation be Einstein–Lovelock (for a given order *N*, all terms $<N$ are also included) or pure Lovelock (only one *N*th order term plus the cosmological constant, which is the 0th order term)? It has been shown that a pure Lovelock equation has the unique distinguishing property that vacuum for static spacetime in all odd $D=2N+1$ dimensions being vanishing in *N*th order Ricci in a kinematic sense implies the corresponding Riemann zero [5–8]. For $N=1$ Einstein gravity, it is kinematic in $D=3$, and it becomes dynamic in the next even dimension $D=4$. For a pure Lovelock equation, this is the unique feature for odd $D=2N+1$ and even $D=2N+2$ dimensions. What order of *N* should be involved in gravitational dynamics is determined by the dimension of spacetime. For example, for $D=3, 4$ it is the $N=1$ Einstein equations (from the Einstein–Hilbert Lagrangian), for $D=5, 6$, it is $N=2$ Gauss–Bonnet, and so on.

Static vacuum solutions are the simplest and most effective tools for probing a new gravitational setting. Beginning with the E–GB black hole solution by Boulware and Deser [9] and independently by Wheeler [10], and its generalization to the general Lovelock case [10–13], all these black holes had horizons with a spherical topology having constant curvature. The next order of generalization was to seek a more general horizon topology of the product of maximally symmetric spaces for Einstein space horizons. Note that the product space no longer remains maximally symmetric; however, its Riemann curvature is covariantly constant.^1^ For an Einstein space, note that $W_{abcd;e} = R_{abcd;e}$, and hence now Weyl curvature is covariantly constant, which for maximally symmetric Riemann is zero. We would therefore term the product space horizon a Weyl constant space. The first interesting solution with this generalization was obtained for an E–GB black hole by Dotti and Gleiser [14] with the horizon space being a Weyl constant Einstein space, as realized by the product of two spheres. This is the case we will concern ourselves with in this paper. The measure of Weyl constancy is expressed as the square of the Weyl curvature, which makes a non-trivial contribution to the gravitational potential of the hole. In this paper we shall discuss solutions for which the horizon is a product of two spheres. It turns out that for the Einstein–Hilbert case for a gravitational potential of the hole, neither does it matter whether two spheres are of equal curvature (and dimensionality) or not, nor whether the topology is of one or two spheres. On the other hand, for the GB case, they have to be of equal curvature and dimensionality. This is because in the latter case the Riemann tensor is directly involved in the equation, while in the former it is only the Ricci tensor, and that is why the latter is more restrictive. In other words, for an Einstein black hole, the horizon space need not even be an Einstein space, while for the GB and higher order Lovelock case it always has to be an Einstein space. There has been a spurt of activity in studying various aspects of Dotti–Gleiser black holes in terms of its uniqueness, thermodynamics, and stability by various authors [15–17].

There is yet another motivation for this paper. The study of spaces with some rotational symmetries in general relativity has been strongly motivated by the property that it provides a rich spectrum of different phases of black objects with distinct topologies for horizons; see for a review [18]. The simplest realization of it is provided by the usual 4-dimensional Schwarzschild black hole with an extra dimension of radius *L* added. If the Schwarzschild radius is much smaller than the radius of the extra dimension, it would resemble the 5-dimensional Schwarzschild black hole with horizon topology $S^3$. On the other hand if the black hole radius is bigger than the extra dimension radius, it would describe a black string with horizon topology $S^2 \times S^1$. This means there does occur a local change in horizon topology depending upon the radius of the horizon being smaller or larger than the compact dimension. This is a purely kinematic feature. It turns out that this change is brought about [19], see also [20], through a cone over $S^2\times S^2$ (for brevity, we shall term it the topology of two spheres) by seeking a Ricci flat metric for the cone. This construction could as well be looked upon as a solid angle deficit for each $S^2$. Note that the angle deficit describes a cosmic string for which the Riemann curvature vanishes, while the solid angle deficit for which the Riemann curvature is non-zero describes a global monopole [21]. The interesting question that arises is whether the contribution of the solid angle deficit of one sphere exactly cancels that of the other, giving rise to Ricci flat space. It is remarkable that this is precisely what happens for Dotti–Gleiser black holes [14] with the two spheres topology. The topology of two spheres harbors a static black hole with constant Ricci (Einstein space) but non-constant Riemann curvature horizon.

In this paper, we would like to study the more general case of $S^{d_1}\times S^{d_2}$ for $\Lambda$-vacuum solutions of the Einstein, Gauss–Bonnet (GB) and Einstein–Gauss–Bonnet (E–GB) equations. That is, a ($d=d_1+d_2+2$)-dimensional spacetime harbors a static black hole with topology of two spheres $S^{d_1} \times S^{d_2}$ for Einstein and $S^{d_0} \times S^{d_0}$ with $d_1=d_2=d_0$ for GB and E–GB gravity. In the latter case we show that the horizon space $S^{d_0} \times S^{d_0}$ has constant Weyl curvature. One of the new features of these GB and E–GB black holes is that there may occur a non-central naked singularity which could, however, be avoided by prescribing a range for the black hole mass in terms of a given $\Lambda$. It is noteworthy that the presence of positive $\Lambda$ is therefore necessary for the existence of these black holes for GB gravity. That is, $\Lambda$ plays a very critical role in this setting as is the case for the stability of a pure Lovelock black hole, where it renders an otherwise unstable black hole stable [22].

Note that we obtain the solutions using a technique different from that of [14]. Starting from the action principle we proceed in two steps. First we perform a consistent truncation together with a dimensional reduction of the Lagrangian, ending up with a reduced Lagrangian with four mechanical degrees of freedom (instead of field theoretical ones). This Lagrangian describes static metrics with $\mathbf{SO(n) \times SO(n)}$ symmetry. In the second step we introduce an ansatz concerning the radial variable for the spheres, and the problem becomes an equation for a single degree of freedom. Then, as is the case for a general Lovelock vacuum equation in spherical symmetry, it all reduces to an algebraic equation.

The paper is organized as follows: We begin with an Einstein black hole and set up the general framework of consistent truncation for solving gravitational equations. We thus obtain black hole solutions by this alternative method. It is then followed by the general setting of black hole solutions with a horizon consisting of product spaces of constant curvature. We study various physical features of these black holes including the prescription of the allowed mass range, thermodynamics, and thermodynamical stability. Next we use the same truncation technique to find solutions of the Einstein, GB, and E–GB cases with two spheres of constant curvature, and we obtain the generalized Nariai metric [23, 24]. We end with a discussion.

## Einstein black hole

We begin with the general static metric of a spacetime with two spheres topology $R^2\times S^{d_1}\times S^{d_2}$, which is written as follows:

1 $$\begin{aligned} \mathrm{d}s^2 = -A(r)\,\mathrm{d}t^2 + B(r)\,\mathrm{d}r^2 + C(r)\,\mathrm{d} S^2_{(d_1)}+ D(r)\,\mathrm{d} S^2_{(d_2)}, \end{aligned}$$

in $d=d_1+d_2+2$ dimensions. We use the notation of indices (0, 1) for (*t*, *r*), $(a,b,\ldots )$ for the angular coordinates of the first sphere $S^{d_1}$ and $(a',b',\ldots )$ for those of the second sphere $S^{d_2}$. We keep the four functions *A*(*r*), *B*(*r*), *C*(*r*), *D*(*r*) as the unknown variables, which is a consistent truncation ansatz. It means that the direct substitution of (1) into the Lagrangian will give the same equations of motion (EOM) (from the truncated Lagrangian) as if directly substituted into the EOM of the original Lagrangian (see details concerning consistent truncations in [25, 26]).

Under this generic ansatz, the only nonvanishing components of the Riemann tensor are

2 $$\begin{aligned} {R_{01}}^{01}= & {} \frac{A(r) A'(r) B'(r)+B(r) (A'(r)^2-2 A(r) A''(r))}{4 A(r)^2 B(r)^2}\nonumber \\=: & {} L(0,1),\nonumber \\ {R_{0a}}^{0a}= & {} -\frac{A'(r) C'(r)}{4 A(r) B(r) C(r)}=:L(0,a),\nonumber \\ {R_{1a}}^{1a}= & {} \frac{C(r) B'(r) C'(r)\!-\!2 B(r) C(r) C''(r)\!+\!B(r) C'(r)^2}{4 B(r)^2 C(r)^2}\nonumber \\ {}=: & {} L(1,a), \end{aligned}$$

3 $$\begin{aligned} {R_{ab}}^{ab}= & {} \frac{1}{C(r)}-\frac{C'(r)^2}{4 B(r) C(r)^2}=:L(a,b),\quad (a\ne b)\nonumber \\ {R_{0a'}}^{0a'}= & {} -\frac{A'(r) D'(r)}{4 A(r) B(r) D(r)}=:L(0,a'),\nonumber \\ {R_{1a'}}^{1a'}= & {} \frac{D(r) B'(r) D'(r)\!-\!2 B(r) D(r) D''(r)\!+\!B(r) D'(r)^2}{4 B(r)^2 D(r)^2}\nonumber \\=: & {} L(1,a'),\nonumber \\ {R_{a'b'}}^{a'b'}= & {} \frac{1}{D(r)}-\frac{D'(r)^2}{4 B(r) D(r)^2}=:L(a',b'),\quad (a'\ne b') \,,\nonumber \\ {R_{a a'}}^{a a'}= & {} -\frac{C'(r) D'(r)}{4 B(r) C(r) D(r)}=:L(a,a'). \end{aligned}$$

The Einstein–Hilbert (EH) Lagrangian (with the cosmological constant term) $\sqrt{-g}( R - 2 \Lambda )$, for the metric (1), reads as follows:

4 $$\begin{aligned} L_{\mathrm{EH}}= & {} \sqrt{-g}(2\, L(0,1)+ 2\, d_1(L(0,a)+L(1,a))\nonumber \\&+2\, d_2(L(0,a')+L(1,a')) \nonumber \\&+ d_1(d_1-1)L(a,b) + d_2(d_2-1)L(a',b')\nonumber \\&+ 2\, d_1d_2 L(a,a')- 2 \Lambda ), \end{aligned}$$

where the density factor $\sqrt{-g}$ is (up to the volume of the spheres, which here is irrelevant)

$$\begin{aligned} \sqrt{-g}\rightarrow \sqrt{A(r) B(r)} C(r)^{\frac{d_1}{2}}D(r)^{\frac{d_2}{2}}. \end{aligned}$$

It is well known that the null energy condition and the fact that the radial photon experiences no acceleration [27] require $B(r)=\frac{1}{A(r)}$, and we set $C(r) =\frac{ r^2}{ k_1}, D(r)=\frac{ r^2}{k_2}$ where $k_1, k_2$ are constants.

The metric thus takes the form

5 $$\begin{aligned} \mathrm{d}s^2 = -A(r)\,\mathrm{d}t^2 + \frac{1}{A(r)} \mathrm{d}r^2 + \frac{ r^2}{ k_1}\,\mathrm{d} S^2_{(d_1)} +\frac{ r^2}{ k_2}\,\mathrm{d} S^2_{(d_2)}. \end{aligned}$$

The constants $k_i$ are fixed as $k_i=\frac{d-3}{d_i -1}$ by solving the EOM for (4) for $A(r)=1$, by which one obtains the results already given in [19]. It turns out that the EOM for the truncated Lagrangian (4) ultimately reduces to a single first order differential equation, given by

6 $$\begin{aligned} \frac{\mathrm{d}}{\mathrm{d}\,r}\left( r^{d-3}\,(1-A(r))- \frac{2\Lambda }{(d-1)(d-2)} r^{d-1}\right) =0. \end{aligned}$$

This readily solves to give the solution

7 $$\begin{aligned} A = 1- \frac{2\Lambda }{(d-1)(d-2)}r^2 - \frac{M}{r^{d-3}}, \end{aligned}$$

where *M* is an integration constant proportional to mass of the configuration as it produces potential in *d* dimension. This is simply because the gravitational field is radially symmetric, though spacetime is not spherically symmetric, for the two spheres horizon topology. It is the Schwarzschild–de Sitter metric potential. Of course it is not Schwarzschild–de Sitter spacetime, as it does not have maximal symmetry when $M=0$. Henceforth all through our discussion by the dS/AdS approaches we generally mean the form of the metric potential to be dS/AdS, and not the entire spacetime. Thus we have the static black hole metric:

8 $$\begin{aligned} \mathrm{d}s^2= & {} -\left( 1- \frac{2\Lambda }{(d-1)(d-2)}r^2 - \frac{M}{r^{d-3}}\right) \,\mathrm{d}t^2 \nonumber \\&+ \frac{1}{\left( 1- \frac{2\Lambda }{(d-1)(d-2)}r^2 - \frac{M}{r^{d-3}}\right) }\,\mathrm{d}r^2\nonumber \\&+ \left( \frac{d_1-1}{d-3}\right) \, r^2\,\mathrm{d} S^2_{(d_1)}+ \left( \frac{d_2-1}{d-3}\right) \, r^2\,\mathrm{d} S^2_{(d_2)}, \end{aligned}$$

with $d_1>1, d_2>1$. (Other cases will be dealt with in the next two subsections.) Notice that the constant coefficient before $\mathrm{d}S^2$ indicates a solid angle deficit which depends upon the dimension of the sphere. The metric (8) describes a black hole with the horizon topology $S^{d_1}\times S^{d_2}$. Note that for $\Lambda =M=0$ the spacetime is not Minkowski because of the solid angle deficits which produce a non-zero Riemann curvature as could be seen from the Kretschmann scalar, $K=R_{\mu \nu \rho \sigma }R^{\mu \nu \rho \sigma }$ which reads

9 $$\begin{aligned} K= & {} \frac{d_1 d_2 (d-4) (d-3)}{2 (d_1-1) (d_2-1)}\frac{1}{r^4}+\left( 1+\frac{2}{d-1}-\frac{2}{d-2}\right) \Lambda ^2\nonumber \\&+ \frac{1}{4} (d-3) (d-2)^2 (d-1) \frac{M^2}{r^{2(d-1)}}. \end{aligned}$$

Clearly it is non-zero when $\Lambda =M=0$ and the spacetime has a singularity at $r=0$. However, it has a weaker divergence ($~1/r^4$) as compared to the black hole ($1/r^{2(d-1)}$). When $\Lambda$ and *M* vanish, the solution coincides with the one proposed in [19], see also [20], as the mediator solution in some topology changing transitions in the space of higher dimensional black hole solutions. Note that the black hole potential, $M/r^{d-3}$, remains unaltered by the topology of two spheres (i.e. $SO(d_1) \times SO(d_2)$ symmetry of the metric). It only rescales $\Lambda$, for the rest it makes no difference at all. Thus for Einstein gravity, the black hole solution is neutral to the product topology; i.e. it does not matter whether it is $S^{d_1}\times S^{d_2}$ or simply $S^{d_1+d_2}$.

### Black string and brane

For $d_1=1$, it turns out that the solution cannot accommodate $\Lambda$ because the one sphere (circle) has no intrinsic curvature. Instead we have the well-known solution

10 $$\begin{aligned} \mathrm{d}s^2= & {} -\left( 1- \frac{M}{r^{d-3}}\right) \,\mathrm{d}t^2 + \frac{1}{\left( 1- \frac{M}{r^{d-3}}\right) }\,\mathrm{d}r^2+ \mathrm{d}z^2\nonumber \\&+ r^2\,\mathrm{d} S^2_{(d-3)}, \quad (z\ \mathrm{periodic}). \end{aligned}$$

This is the uniform black string solution in which a flat direction is added to a Schwarzschild black hole [28].

On the other hand if we take one of the spheres to be of constant curvature and the other without solid angle deficit, then it would give a black brane with the metric

11 $$\begin{aligned} \mathrm{d}s^2 = -A(r)\,\mathrm{d}t^2 + \frac{1}{A(r)}\,\mathrm{d}r^2 + r^2\,\mathrm{d} S^2_{(d_1)}\pm \frac{1}{k}\,\mathrm{d} \Sigma ^2_{(d_2)}, \end{aligned}$$

where

12 $$\begin{aligned} A(r)= & {} 1- \frac{({d_2}-1) }{{d_1}+1}\,k\,r^2-\frac{M}{r^{d_1-1}},\nonumber \\\Lambda= & {} \frac{1}{2} ({d_2}-1)({d_1}+{d_2})\,k. \end{aligned}$$

Here $\Sigma$ is a space of constant curvature, a sphere ($k>0$), a hyperboloid ($k<0$) or flat ($k=0$). That is, a constant curvature space is added to a Schwarzschild–de Sitter/AdS black hole in $d=d_1+2$ dimensions and hence it may be taken as a uniform black brane [28].

### Generalized Nariai metric

For $M=0$ in Eq. (12), we have a product of two spaces of constant curvature, $R^{d_1+2}\times S^{d_2}$, it is a generalized Nariai solution [5, 23, 24] of $R_{ab}=\Lambda g_{ab}$. Let us consider the product of two constant curvature spaces, $R^2 \times S^2$. If the curvatures are equal, it is a Nariai solution of $R_{ab}=\Lambda g_{ab}$, on the other hand, if they are equal and opposite in sign, then it is an Einstein–Maxwell solution [30, 31] describing a gravitational field of a uniform electric field. Contrary to general behavior of such spacetimes, the former is conformally non-flat, while the latter is conformally flat. Note that both product spaces are of the same dimension, while in our case, they are of different dimensions, $R^{d_1+2}\times S^{d_2}$, and that is why we call it a generalized Nariai metric. For $d_2=d_1=2$ where $R^4\times S^2$, the generalized Nariai metric would take the form

13 $$\begin{aligned} \mathrm{d}s^2 = -\left( 1-\frac{\Lambda }{6} r^2\right) \,\mathrm{d}t^2+ & {} \frac{1}{1-\frac{\Lambda }{6} r^2}\,\mathrm{d}r^2\nonumber \\+ & {} r^2\,\mathrm{d}S^2_2 + \frac{2}{\Lambda }\,\mathrm{d} \Sigma ^2_{2}. \end{aligned}$$

It is a product of 4-dimensional dS and a two sphere of constant curvature. In fact one can have any *n*-dimensional dS with any *m* sphere of constant curvature to give a generalized Nariai metric.

Thus the two spheres ansatz we have considered encompasses black objects like hole, string, brane, and generalized Nariai solutions.

## GB black hole

We can use the same method as above to write down the reduced – consistently truncated – GB Lagrangian in terms of the variables *A*(*r*), *B*(*r*), *C*(*r*), *D*(*r*) given in (1) and with the use of Eq. (3). So we start by considering the GB Lagrangian (with cosmological constant)

14 $$\begin{aligned} L_{\mathrm{GB}}=\sqrt{-g}(- 2 \Lambda + {R}^2 -4 {R_\mu }^\nu {R_\nu }^\mu + {R_{\mu \nu }}^{\rho \sigma }{R_{\rho \sigma }}^{\mu \nu }) \end{aligned}$$

and truncate it by implementing the ansatz (1) into it, analogously to (4). Since it is not particularly illuminating, the resulting truncated Lagrangian is given in detail in the “Appendix”.

We begin with the metric (5),

15 $$\begin{aligned} \mathrm{d}s^2 = -A(r)\,\mathrm{d}t^2 + \frac{1}{A(r)}\,\mathrm{d}r^2 + \frac{r^2}{k_1}\,\mathrm{d} S^2_{(d_1)}+ \frac{r^2}{k_2}\,\mathrm{d} S^2_{(d_2)}, \end{aligned}$$

with $d_1>1, d_2>1$. It turns out that we are able to find analytic solutions when the two spheres have the same dimension $d_1=d_2=:d_0$. This means $d=d_1+d_2+2=2(d_0+1)$ and $k_1=k_2=k = \frac{2 d_0-1}{d_0-1}$. Let us define

16 $$\begin{aligned} \Psi (r):= 1-A(r), \end{aligned}$$

then EOM [from the truncated Lagrangian (53)] again becomes a single first order differential equation,

17 $$\begin{aligned}&\frac{\mathrm{d}}{\mathrm{d}\,r}\left( r^{2 d_0-3}\Big ( \psi ^2 +\frac{d_0}{(d_0-1)^2 (2 d_0-3)}\Big )\right. \nonumber \\&\left. \quad - \frac{\Lambda r^{2 d_0+1}}{2 (2 d_0+1)(2 d_0-1)( d_0-1)d_0}\right) =0. \end{aligned}$$

It integrates to give

18 $$\begin{aligned}&A(r)=1 \pm \nonumber \\&\sqrt{-\frac{d_0}{(d_0-1)^2 (2 d_0-3)}+\frac{ r^4 \Lambda }{2(2 d_0+1)(2 d_0-1)( d_0-1)d_0 } +\frac{M }{ r^{2 d_0-3}}},\nonumber \\ \end{aligned}$$

where *M* is an integration constant proportional to mass of the configuration. It represents a black hole in an asymptotically dS spacetime. This is a new black hole solution with two spheres topology in GB gravity. We would choose negative sign so as (18) to accord asymptotically to Schwarzschild–de Sitter spacetime while positive sign will make gravity repulsive leading to naked singularity.

It is obvious that the solution cannot admit $M=\Lambda =0$ limit and clearly reality of the metric as well as existence of horizons would prescribe a bound on mass of the black hole in relation to $\Lambda$. That is what we next consider.

### Reality and physical bounds for (18)

Since in absence of black hole ($M=0$), $\Lambda$ must be positive, and hence we shall take both *M* and $\Lambda$ to be always non-negative. For concreteness let’s set $d_0=2$ which means we are considering 6-dimensional black hole solution. Further for simplicity we define $\tilde{\Lambda }=\frac{\Lambda }{15}$, we write the solution (18) for $d_0=2$ as

19 $$\begin{aligned} A(r)=1-\sqrt{-2+\frac{1}{4}\tilde{\Lambda }r^4+\frac{M}{r}}, \end{aligned}$$

where $\tilde{\Lambda }$ and *M* are taken to be non-negative.

Clearly for reality of the solution the discriminant should be $\ge 0$ as well as $A\ge 0$ for the existence of black hole horizon. Both these conditions should hold good simultaneously which means

20 $$\begin{aligned} 2\le h(r)\le 3 \end{aligned}$$

where $h(r) := \frac{1}{4}\tilde{\Lambda }r^4+\frac{M}{r}$. The lower bound guarantees non-negativity of the discriminant, while the upper bound ensures the existence of horizons bounding a regular region of spacetime. The function *h*(*r*) has a single minimum at

21 $$\begin{aligned} r_0=\Big (\frac{M}{\tilde{\Lambda }}\Big )^\frac{1}{5} \end{aligned}$$

and

22 $$\begin{aligned} h(r_0) = \frac{5}{4} \tilde{\Lambda }^\frac{1}{5} M^\frac{4}{5}. \end{aligned}$$

As will be discussed below, for the physical viability of a black hole we must have $2\le h(r_0)\le 3$, which implies

23 $$\begin{aligned} 2\le \frac{5}{4} \tilde{\Lambda }^\frac{1}{5} M^\frac{4}{5}\le 3, \end{aligned}$$

or, equivalently,

24 $$\begin{aligned} \Big (\frac{8}{5}\Big )^5\le \tilde{\Lambda }M^4\le \Big (\frac{12}{5}\Big )^5. \end{aligned}$$

**Fig. 1:**
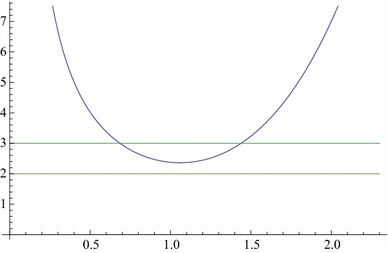
Plot of *h*(*r*) when $(\frac{8}{5})^5< \tilde{\Lambda }M^4< (\frac{12}{5})^5$. The *horizontal lines* are $y=3$ and $y=2$, The intersections of *h*(*r*) with the *upper line* $y=3$ define the horizons, black hole, and cosmological cases

The lower bound is given by the discriminant being non-negative, while the upper bound is given by the existence of horizons. The horizons are given by $h(r)=3$, which is a fifth degree equation and can have two positive roots giving two horizons, lower ($r_-$) and upper ($r_+$), respectively, for the black hole and cosmological, dS-like, case, as shown in Fig.  1.

**Fig. 2:**
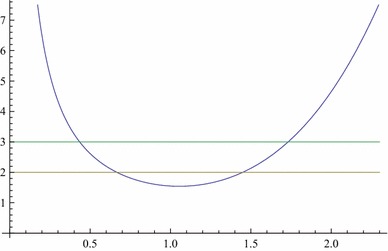
Plot of *h*(*r*) when $\tilde{\Lambda }M^4< (\frac{8}{5})^5$. The *horizontal lines* are $y=3$ and $y=2$, the intersections of *h*(*r*) with the *lower line* $y=2$ determine the singularities. The critical case $\tilde{\Lambda }M^4= (\frac{8}{5})^5$ (not depicted here) means tangency with the *lower line*, and there is no singularity

There is an unusual feature of this class of black holes: there occurs a curvature singularity at vanishing of the discriminant, $h(r)=2$. As a matter of fact the Ricci scalar for the GB black hole (19) is given by

$$\begin{aligned} R\!=\!\frac{70 M^2\!+\!6 M r (15 \tilde{\Lambda }r^4\!-\!56)\!+\!r^2 (3 \tilde{\Lambda }r^4\!-\!8) (5 \tilde{\Lambda }r^4\!-\!48)}{r^4 \left( \frac{4 M}{r}+\tilde{\Lambda }r^4-8\right) ^{3/2}}, \end{aligned}$$

which clearly diverges for $h(r_1)=2$ unless the numerator also vanishes at $r_1$. The numerator and denominator both vanish simultaneously only for $\tilde{\Lambda }M^4=(\frac{8}{5})^5$ at $r_1=\frac{5\, M}{8}= (\frac{8}{5\,\tilde{\Lambda }})^{\frac{1}{4}}$, making *R* finite. This marks the limiting minimum for the black hole mass at which $r_1$ becomes the minimum $r_0$, as shown in Fig. 2.

The remarkable property of this class of black holes is therefore the existence of an extremal value for the mass which is a minimum. Below this minimum there occur two naked singularities for $r>0$ given by $r^5 -8r/{\tilde{\Lambda }} +4M/{\tilde{\Lambda }} = 0$. This is in addition to the usual central singularity at $r=0$. It is the case that the latter is always covered by a horizon for the black hole, while for the former the only option is not to let them occur. This is precisely what the above bounds on the mass for a given $\Lambda$ do as shown in Figs. 1 and 2. This requires both $\Lambda ,\,M$ to be non-zero. It can easily be seen that either of them being zero makes a non-central ($h(r)=2$) singularity naked. It is not only so that it remains naked for $\Lambda < 0, \, M>0$ and hence $\Lambda$ must always be positive. This is a new property of this class of black holes. In this setting, a black hole can thus exist only in an asymptotically de Sitter spacetime. That is, the presence of a positive $\Lambda$ is critical for the existence of a black hole. Very recently a similar result has also been obtained for the stability of pure Lovelock black holes [22], where $\Lambda$ makes an otherwise unstable black hole stable.

## E–GB black hole

Now we consider the E–GB Lagrangian

25 $$\begin{aligned} {L_{\mathrm{E-GB}}}= & {} \sqrt{-g} (-2 \Lambda + \alpha _1{R} + \alpha _2\,({R}^2 -4 {R_\mu }^\nu {R_\nu }^\mu \nonumber \\&+ {R_{\mu \nu }}^{\rho \sigma }{R_{\rho \sigma }}^{\mu \nu }) ), \end{aligned}$$

with separate parameters $\alpha _1$ and $\alpha _2$, so we can recover the GB and EH cases as limits with either parameter vanishing. The consistent truncation of (25) under (3) is given by (4) and in (53) (see the “Appendix”).

Again we consider the specific metric

26 $$\begin{aligned} \mathrm{d}s^2 = -A(r)\,\mathrm{d}t^2 + \frac{1}{A(r)}\,\mathrm{d}r^2 + \frac{d_0-1}{d-3}r^2(\mathrm{d} S^2_{(d_0)}+ \mathrm{d} S^2_{(d_0)}). \end{aligned}$$

Now the EOM for the variable *A*(*r*) takes the form

27 $$\begin{aligned}&\frac{\mathrm{d}}{\mathrm{d}\,r}\left( \alpha _1\, r^{2 d_0-1}\Big (\frac{1}{2(2 d_0^2-3 d_0+1)}\Big ) \Psi \right. \nonumber \\&\quad +\left. \alpha _2\, r^{2 d_0-3}\Big ( \Psi ^2 +\quad \frac{d_0}{(d_0-1)^2 (2 d_0-3)}\Big )\right. \nonumber \\&\quad \left. - \frac{\Lambda r^{2 d_0+1}}{2(2 d_0+1)(2 d_0-1)( d_0-1)d_0}\right) =0, \end{aligned}$$

where $\Psi (r)$ has been defined in (16). This integrates to give the solution

28 $$\begin{aligned} A(r)= & {} 1+\frac{\alpha _1 r^2}{4 (2 d_0-1)(d_0-1)\alpha _2 }\nonumber \\&-\Big (\!-\frac{d_0}{(d_0\!-\!1)^2 (2 d_0\!-\!3)} \!+\!\frac{\alpha _1^2 r^4}{4^2 (2 d_0\!-\!1)^2(d_0\!-\!1)^2 \alpha _2^2}\nonumber \\&+ \frac{ \Lambda r^4 }{2(2 d_0+1)(2 d_0-1)( d_0\!-\!1)d_0\, \alpha _2 }+\frac{M}{\alpha _2\, r^{2 d_0-3}}\Big )^\frac{1}{2},\nonumber \\ \end{aligned}$$

where we have chosen the negative sign before the radical for the same reason as for GB case.

In the limit $\alpha _2\rightarrow 0$ we recover

29 $$\begin{aligned} \lim _{\alpha _2\rightarrow 0}A(r)= 1- \frac{\Lambda }{(2 d_0^2+d_0)\,\alpha _1}r^2- \frac{2 M ((2 d_0-3) d_0+1)}{\alpha _1\,r^{2 d_0-1}}, \end{aligned}$$

the corresponding Schwarzschild–de Sitter solution (8) for $d_0=\frac{d}{2}-1$ and $\alpha _1=1$ with an appropriate redefinition of the mass parameter, *M*.

Let us now set $d_0=2$ and then

30 $$\begin{aligned} A(r)=1+\frac{\alpha _1}{12\, \alpha _2}r^2 -\sqrt{-2+\left( \frac{ \alpha _1^2}{(12\,\alpha _2)^2}+\frac{ \Lambda }{60\, \alpha _2}\right) r^4+ \frac{M}{\alpha _2\,r}}. \end{aligned}$$

It is interesting to compare this solution with the solution with one sphere topology,

31 $$\begin{aligned} \mathrm{d}s^2 = -A(r)\,\mathrm{d}t^2 + \frac{1}{A(r)}\,\mathrm{d}r^2 + r^2\,\mathrm{d} S^2_{(d-2)}, \end{aligned}$$

with

32 $$\begin{aligned} A(r)_{\!{}_{\mathrm{one}\ \mathrm{sphere}}}\!=\!1+\frac{\alpha _1}{12\, \alpha _2}r^2 -\sqrt{\left( \frac{ \alpha _1^2}{(12\,\alpha _2)^2}+\frac{ \Lambda }{60\, \alpha _2}\right) r^4\!+\!\frac{M}{\, \alpha _2\,r}}. \end{aligned}$$

It is indeed the same as the above without $-2$ under the radical. Note that the former is not asymptotically flat for $\Lambda =0$, while the latter is asymptotically flat, i.e. Minkowski. The other difference of course is that in the former metric, each sphere has a solid angle deficit which cancels out the other to give a $\Lambda$-vacuum spacetime. This is true more generally for $d=2d_0+2$ where the former will have $-\frac{d_0}{(d_0-1)^2 (2 d_0-3)}$ under the radical, while the latter would be free of it.

### Physical bounds for (28)

Let us rewrite (28) as

33 $$\begin{aligned} A(r)= & {} 1+D r^2 -\sqrt{f(r)},\nonumber \\ f(r)= & {} -C +\frac{1}{4} E^{2 d_0+1} r^4+\frac{1}{2 d_0-3}B^{2 d_0+1}\frac{1 }{r^{2 d_0-3}}, \end{aligned}$$

with

34 $$\begin{aligned} E^{2 d_0+1}= & {} \frac{\alpha _1^2 }{4 (2 d_0-1)^2(d_0-1)^2 \alpha _2^2}\nonumber \\&+\frac{\Lambda }{2(2 d_0+1)(2 d_0-1)( d_0-1)d_0\,\alpha _2 }\nonumber \\ B^{2 d_0+1}=: & {} (2 d_0-3)\frac{M}{\alpha _2}\nonumber \\ C= & {} \frac{d_0}{(d_0-1)^2 (2 d_0-3)}\nonumber \\ D= & {} \frac{\alpha _1 }{4 (2 d_0-1)(d_0-1)\alpha _2 }. \end{aligned}$$

Note that since their exponent is odd, the signs of *E* and *B* are those of their respective right hand side in the definition.

Let us consider the case $\alpha _1>0$ and $\alpha _2>0$ (the same sign for the EH and GB coefficients is required for the theory to be ghost free [9]) as well as $\Lambda >0$ and $M>0$, which imply $E>0,\,B>0$. Note from (34) that there is a window for negative $\Lambda$ and still keeping $E>0$.

The minimum for *f* is attained at $\displaystyle r_0= \frac{B}{E}$. Following the same lines as before (see Sect. 3.1), we obtain the bounds as follows:

35 $$\begin{aligned} C\,\le \,\frac{2 d_0+1}{4(2 d_0 -3)}E^{2 d_0-3} B^4\,\le \,C+\left( 1+D\left( \frac{B}{E}\right) ^2\right) ^2 \end{aligned}$$

where *C*, given in (34), is a positive quantity determined by the spacetime dimension. Since *E*, *B*, *D* are positive, it is clear, for given $\alpha _1,\alpha _2$, that there exist a range of values for *E* and *B* (i.e. for $\Lambda$ and *M*) fulfilling the bounds (35). Thus as before a non-central curvature singularity at the vanishing of the radical could be avoided by a suitable prescription on the black hole mass for given $\Lambda$.

## Thermodynamics of black holes

We will use the notation of Eq. (34) and of course we assume that the conditions given in Eq. (35) hold true, which guarantee the existence of horizons. Let us denote the black hole horizon by $r_h$. Of course, $r_h$ and *M* could be traded for each other, just by requiring the function $M(r_h)$ to keep $A(r_h)=0$, while varying $r_h$. The entropy is calculated from the first law of thermodynamics by following the standard procedure.

We write the identity

36 $$\begin{aligned} A(r,M(r))=0 \end{aligned}$$

as an implicit equation for the function *M*(*r*) introduced above; therefore, we have the identity

37 $$\begin{aligned} A'(r,M(r)) + \frac{\partial A}{\partial M}|_{A=0}\,M'(r)=0, \end{aligned}$$

where $A'(r,M(r))$ denotes the derivative relative to the first argument *r*. Employing the Euclidean method (with periodic time to eliminate a conical singularity), we identify $A'(r,M(r))$ as the Hawking temperature $\displaystyle T(r) =\frac{A'(r,M(r))}{4\pi }$.

Thus we have

38 $$\begin{aligned} \frac{M'(r)}{T} = - 4\pi \frac{1}{\frac{\partial A}{\partial M}|_{A=0}} = 8\pi \alpha _2(1+D r^2) r^{2d_0-3}, \end{aligned}$$

and we can compute the entropy by integrating the first law,

39 $$\begin{aligned} S= & {} \int \frac{\mathrm{d} M}{T} = \int \frac{M'(r)}{T(r)} \mathrm{d}r = 8\pi \alpha _2\int (1+D r^2) r^{2d_0-3} \mathrm{d}r \nonumber \\= & {} 8\pi \alpha _2\, r_h^{2 d_0}\Big (\frac{1}{(2 d_0-2)r_h^2} + \frac{D }{2 d_0}\Big ), \end{aligned}$$

where we have assumed that the entropy vanishes when the horizon shrinks to zero.

Of course, the parameter *M* used in our derivation is identified with the mass except for an overall factor that will depend on the dimension of the spacetime and linearly on the area of two spheres at unit radius. With this factor installed we get

40 $$\begin{aligned} S\simeq & {} \mathbf{A} \times \Bigg (\frac{\alpha _2}{(2 d_0-2)r_h^2} + \alpha _2 \frac{D }{2 d_0}\Bigg )\nonumber \\= & {} \mathbf{A}\times \Bigg (\frac{\alpha _2}{(2 d_0-2)r_h^2}+ \frac{\alpha _1}{8 d_0 (2 d_0-1)(d_0-1)}\Bigg )\nonumber \\ {}= & {} \mathbf{A}\times \Bigg (\frac{\bar{\alpha }_2}{r_h^2}+ \bar{\alpha }_1 \Bigg ), \end{aligned}$$

where $\mathbf A$ denotes the horizon area and $\bar{\alpha }_1 = \alpha _1/(8d_0(2d_0-1)(d_0-1))$ and $\bar{\alpha }_2 = \alpha _2/(2d_0-2)$.

The temperature, in terms of $r_h$, is

41 $$\begin{aligned} T= & {} \frac{1}{ 8\pi \,(1\!+\!D r_h^2)r_h}\Bigg (\! (1+D r_h^2) (2 d_0-3 +(2 d_0+1)D r_h^2) \nonumber \\&+\,(2 d_0\!-\!3)C-\frac{2 d_0+1}{4}E^{2 d_0+1} r_h^4 \!\Bigg ). \end{aligned}$$

For instance, for the pure GB case ($\alpha _2=1, \alpha _1=0 \,(\Rightarrow D=0)$) and $d_0=2$ ($d=6$), we obtain

42 $$\begin{aligned} T= \frac{1}{2\pi }\left( -\frac{3}{r_h}+\frac{5}{4}\frac{M}{r_h^{2}} \right) ,\qquad S\simeq \frac{ \mathbf{A}}{ r_h^2} \simeq r_h^2 \simeq \mathbf{A}^{\frac{1}{2}}. \end{aligned}$$

It is worth noting that these parameters bear the same universal relation to $r_h$ as established in [32] for a pure Lovelock one sphere topology in the critical dimension $d=2N+2$, here for the $N=2$ case. In particular, for a pure GB black hole, $T= \frac{1}{2\pi }(-\frac{1}{r_h}+\frac{5}{4}\frac{M}{r_h^{2}} )$ and $S=4\pi r_h^2$. Thus the thermodynamics parameters do not distinguish between the one and the two sphere topology, save for numerical factors.

Let us finally discuss the stability of the GB black hole. Here we take $\alpha _1=0$ and $\alpha _2=1$. The temperature obtained above gives, for the GB case ($D=0$),

43 $$\begin{aligned} T =\frac{1}{8\pi \, r_h}\Bigg ( (2 d_0-3)(C +1) -\frac{2 d_0+1}{4}E^{2 d_0+1} r_h^4 \Bigg ), \end{aligned}$$

and for it to be positive we need

44 $$\begin{aligned} \frac{2 d_0+1}{4(2 d_0-3)}E^{2 d_0+1} r_h^4 \le (C +1). \end{aligned}$$

Actually this requirement is more restrictive than the bound determined before (35), which written in terms of $r_0$ (the minimum of *f*) becomes, for the side we are interested in, $\displaystyle \frac{2 d_0+1}{4(2 d_0-3)}E^{2 d_0+1} r_0^4 \le (C +1)$. Since according to our construction $r_0<r_h$, in order to keep *T* positive, we must replace the rhs of the bound (35) by (44), in the $D=0$ case; from $S^3$ to $S^2 \times S^1$ when the compact dimension is smaller than the horizon radius as the radius of the hole increases. Once we set the bounds to have a positive temperature, local thermodynamical stability will correspond to a positive specific heat, $\displaystyle C_e=\frac{\mathrm{d} M}{\mathrm{d} T}$. Using the expressions $M(r_h)$ and $T(r_h)$ we have

$$\begin{aligned} C_e = \frac{M'(r_h)}{T'(r_h)} = \frac{M'(r_h)}{T}\frac{T}{T'(r_h)}, \end{aligned}$$

and we have from (38) $\frac{M'(r)}{T} = 8\pi r^{2d_0-3} >0$,. Since $T>0$, we need $T'(r_h)>0$ for $C_e$ to be positive. But clearly we infer from (43) that $T'(r_h)<0$ and thus our solution is not thermodynamically stable. Since the one sphere (instead of two) case could be obtained by setting the parameter *C* to zero in Eq. (34) [see (30, 32)], it is clear that the instability found here is the same as in the spherically symmetric case.

## Solutions with two spheres of constant curvature

In this section we apply the same consistent truncation method to obtain new solutions to the EH, GB, and E–GB cases with two spheres of constant curvature. The metric is therefore of the form

45 $$\begin{aligned} \mathrm{d}s^2 = -A(r)\,\mathrm{d}t^2 + \frac{1}{A(r)}\,\mathrm{d}r^2 + \frac{d_1\!-\!1}{k}\,\mathrm{d} S^2_{(d_1)}+ \frac{d_2\!-\!1}{k}\,\mathrm{d} S^2_{(d_2)}, \end{aligned}$$

with $d_1>1, d_2>1$ and $k>0$. Note that the constant curvature of each sphere, $\frac{k}{d_i-1}$, is different according to its dimensionality. We are seeking a solution of the $\Lambda$-vacuum equation, and hence (*t*, *r*) space also has to be of constant curvature. That is, it can be dS$_2$ or AdS$_2$. The topology is therefore dS$_2$/AdS$_2\times S^{d_1}\times S^{d_2}$. This is a generalized Nariai spacetime [23, 24].

Clearly $A=1-kr^2$ in the above metric is the Einstein solution and $\Lambda$ is determined as $\Lambda = \frac{1}{2}(d_1+d_2)k >0$.

When $k<0$ there are hyperboloids in place of spheres, and the solution could then be written as

46 $$\begin{aligned} \mathrm{d}s^2= & {} -(1+ |k| r^2)\,\mathrm{d}t^2 + \frac{1}{1+ |k| r^2}\,\mathrm{d}r^2 + \frac{d_1-1}{|k|}\,\mathrm{d} H^2_{(d_1)}\nonumber \\&+ \frac{d_2-1}{|k|}\,\mathrm{d} H^2_{(d_2)}, \end{aligned}$$

with $\Lambda =\frac{1}{2} (d_1+d_2) k<0$ and topology, AdS$_2\times H^{d_1}\times H^{d_2}$. This is a generalized anti-Nariai metric [29]. The case $k\rightarrow 0$ is Minkowski spacetime (the spheres or hyperboloids acquire an infinite radius and become flat), though the metric (46) is no longer convenient to describe such a limit.

For the case of GB, we find solutions for (45) with $A=1-k r^2$ and *k* determined below. The curvatures $k_1$ and $k_2$ are constrained by a third degree polynomial equation,

47 $$\begin{aligned}&({d_1}-1) ({d_2}-1) {k_1}^2 {k_2} (({d_1}-2)^2 ({d_1}+3)\nonumber \\&\quad -2 (({d_1}-3) {d_1}+3) {d_2})\nonumber \\&\quad +({d_1}-1) ({d_2}-1) {k_1} {k_2}^2 (2 {d_1} (({d_2}-3) {d_2}+3)\nonumber \\&\quad -({d_2}-2)^2 ({d_2}+3))\nonumber \\&\quad +(3-{d_1}) ({d_1}-2) ({d_1}-1)^2 {d_1} {k_1}^3\nonumber \\&\quad +({d_2}-3) ({d_2}-2) ({d_2}-1)^2 {d_2} {k_2}^3=0, \end{aligned}$$

with $\Lambda$ (here $\Lambda$ includes a dimensional factor originating with the dimensionality of the GB Lagrangian) given by

48 $$\begin{aligned} \Lambda= & {} \frac{1}{8} (2 ({d_1}-1) {d_1} ({d_2}-1) {d_2} {k_1} {k_2}+({d_1}-3)\nonumber \\&\quad ({d_1}-2) ({d_1}-1) {d_1} {k_1}^2\nonumber \\&+({d_2}-3) ({d_2}-2) ({d_2}-1) {d_2} {k_2}^2) \end{aligned}$$

and

49 $$\begin{aligned} k = \frac{({d_1}-1) {k_1} (({d_1}-3) ({d_1}-2) {k_1}+({d_2}-1) {d_2} {k_2})}{({d_1}-2) ({d_1}-1) {k_1}+({d_2}-1) {d_2} {k_2}}. \end{aligned}$$

For the equal dimension spheres, $d_1=d_2=:d_0$ and $k_1=k_2$, they become

50 $$\begin{aligned} \Lambda= & {} \frac{1}{2} (d_0-1) d_0 ((d_0-3) d_0+3) k^2,\nonumber \\ k= & {} \frac{((d_0-3) d_0+3) }{d_0-1} \, k_1. \end{aligned}$$

We continue with (45) and $A=1-k r^2$. As expected for E–GB $k_1$ and $k_2$ have also to satisfy a third order polynomial equation. We consider the simple case of $d_1=d_2=d_0$ and $k_1=k_2$, and then we obtain

51 $$\begin{aligned} \Lambda = \frac{1}{2} (d_0-1) d_0 k_1 (2\alpha _1+4 ((d_0-3) d_0+3) k_1\alpha _2 ) \end{aligned}$$

and

52 $$\begin{aligned} k = \frac{(d_0-1) (\alpha _1+4 ((d_0-3) d_0+3) k_1 \alpha _2)}{\alpha _1+4 (d_0-1)^2 k_1 \alpha _2} k_1, \end{aligned}$$

which reduces to the EH solution for $\alpha _2\rightarrow 0$ and to GB for $\alpha _1\rightarrow 0$.

These GB and E–GB cases also admit hyperboloids in place of spheres. The constant spheres are spacetimes whereas constant hyperboloids are generalized anti-Nariai spacetimes.

## Discussion

It is well known that the vacuum equation for Einstein as well as for general Lovelock gravity in spherical symmetry ultimately reduces to a single first order equation which is an exact differential [33–38] and hence can be integrated trivially. As a matter of fact, the equation then turns out to be purely algebraic for one sphere topology with $SO(d-2)$ symmetry. Interestingly this feature is carried through even for the topology of two spheres with $SO(d_0)\times SO(d_0)$ symmetry where $d=2(d_0+1)$. In particular, Eqs. (6), (17), and (27) refer, respectively, to Einstein, GB, and E–GB gravity, which yield static black hole solutions. This result obviously raises the question as to whether this feature is also carried over to Lovelock gravity in general. The answer is in affirmative and it would be taken up separately in a forthcoming paper [39].

For a black string, there occurs a local topology change as the radius of the horizon increases from that of a black hole $S^{d_0}$ to that of a black string $S^{d_0-1} \times S^1$. This change is determined through [19] a Ricci flat space over a double cone formed by two spheres with a solid angle deficit. The distinguishing feature of this class of Dotti–Gleiser black holes [14] is that the horizon space is an Einstein space with both Weyl and Riemann curvatures being covariantly constant. Contrary to what is said in the literature following Ref. [14], the space, though, is not maximally symmetric; i.e. Riemann curvature is not given in terms of the metric but its covariant derivative is zero.

This non-zero Weyl curvature makes a non-trivial contribution in the black hole potential which gives rise to non-central naked singularity. For Einstein black hole, the topology is $S^{d_1} \times S^{d_2}$, while for GB and E–GB it is $S^{d_0} \times S^{d_0}$, and further it makes no contribution to the potential for the former. This is because the Einstein–Riemann and Weyl curvatures do not enter into the equation, while they do so for the Lovelock case.

For GB and E–GB black holes, what $SO(n)\times SO(n)$ symmetry entails is the occurrence of an additional non-central curvature singularity which could be let not to occur by a suitable prescription on the black hole mass for a given $\Lambda > 0$ (for an E–GB black hole, a narrow window of negative $\Lambda$ is also permitted). The two extremal limits for the mass are defined by the non-occurrence of a non-central naked singularity (an intersection with the lower line in Fig. 2) and the existence of horizons (an intersection with the upper line in Fig. 1). The range for the mass is given in Eqs. (23) and (33), which ensures the absence of a naked singularity for the GB and E–GB black holes in dS spacetime. Also a non-central singularity cannot be avoided when $M=0$ or $\Lambda \le 0$. Thus $\Lambda$ plays a very critical role in the existence of this class of black holes. This reminds one of the recently obtained result in which $\Lambda$ makes an otherwise unstable pure Lovelock black hole stable by similarly prescribing a range of values for the mass [22].

Further it turns out that black hole thermodynamics does not, however, distinguish between the topology of two spheres and one sphere, as the expressions for the temperature and the entropy of the black hole remain essentially the same. For a pure Lovelock black hole with spherical symmetry, the thermodynamics is universal; i.e. the temperature and the entropy bear the same relation to the radius of the horizon in all odd ($d=2N+1$) and even ($d=2N+2$) dimensions where *N* is the degree of the Lovelock Lagrangian [32]. It is interesting that this universality continues to hold true even for black holes with the topology of two spheres in GB and E–GB gravity.

Finally, let us mention that the technique of the truncated Lagrangians allows us to find new solutions with two spheres of constant curvature (or hyperboloids). They correspond to generalized Nariai (or anti-Nariai for hyperboloids) solutions.

## Appendix: The truncated GB Lagrangian

After some combinatorics, the GB Lagrangian (14) (without the cosmological constant), reduced under (1), becomes, with the notation introduced in (3),

53 $$\begin{aligned}&L_{GB}=\sqrt{-g}(8\, d_1 d_2 L(0,1) L(a,a')\nonumber \\&\quad +4\, (d_1-1) d_1 L(0,1) L(a,b)\nonumber \\&\quad +8\, (d_1-1) d_1 d_2 L(a,a') (L(0,a)+L(1,a))\nonumber \\&\quad +4\, d_1 (d_2-1) d_2 L(a',b') (L(0,a)+L(1,a))\nonumber \\&\quad +8\, d_1 d_2 L(0,a) L(1,a')+4\, (d_1-2) (d_1-1) d_1 L(a,b)\nonumber \\&\quad \times \,(L(0,a)+L(1,a))\nonumber \\&\quad + 8\, (d_1-1) d_1 L(0,a) L(1,a)+8\, d_1 d_2 L(0,a') L(1,a)\nonumber \\&\quad +8\, d_1 (d_2-1) d_2 L(a,a') (L(0,a')+L(1,a'))\nonumber \\&\quad +4\, (d_1-1) d_1 d_2 L(a,b)(L(0,a')+L(1,a'))\nonumber \\&\quad +4\, (d_1-1) d_1 (d_2-1) d_2 L(a,a')^2\nonumber \\&\quad +4\, (d_1-2) (d_1-1) d_1 d_2 L(a,a') L(a,b)\nonumber \\&\quad +4\, d_1 (d_2-2) (d_2-1) d_2 L(a,a') L(a',b')\nonumber \\&\quad +2\, (d_1-1) d_1 (d_2-1) d_2 L(a,b) L(a',b')\nonumber \\&\quad + (d_1-3) (d_1-2) (d_1-1) d_1 L(a,b)^2\nonumber \\&\quad + 4\, (d_2-1) d_2 L(0,1) L(a',b')+4\, (d_2-2) (d_2-1) d_2\nonumber \\&\quad \times \,L(a',b') (L(0,a')+L(1,a'))\nonumber \\&\quad +8 \, (d_2-1) d_2 L(0,a') L(1,a')+ (d_2-3) (d_2-2) \end{aligned}$$

## Footnotes

1 Though in the literature, following Ref. [14] this product space is termed non-constant curvature space, its Riemann curvature is indeed covariantly constant but not maximally symmetric.
